# Mild Conditions for Deuteration of Primary and Secondary Arylamines for the Synthesis of Deuterated Optoelectronic Organic Molecules

**DOI:** 10.3390/molecules191118604

**Published:** 2014-11-13

**Authors:** Anwen M. Krause-Heuer, Nageshwar R. Yepuri, Tamim A. Darwish, Peter J. Holden

**Affiliations:** National Deuteration Facility, Bragg Institute, Australian Nuclear Science and Technology Organisation (ANSTO), Locked Bag 2001, Kirrawee DC, NSW 2232, Australia; E-Mails: anwen.krause-heuer@ansto.gov.au (A.M.K.-H.); nageshwar.yepuri@ansto.gov.au (N.R.Y.); peter.holden@ansto.gov.au (P.J.H.)

**Keywords:** arylamine, deuteration, H/D exchange, neutron reflectrometry, OLED, optoelectronics

## Abstract

Deuterated arylamines demonstrate great potential for use in optoelectronic devices, but their widespread utility requires a method for large-scale synthesis. The incorporation of these deuterated materials into optoelectronic devices also provides the opportunity for studies of the functioning device using neutron reflectometry based on the difference in the scattering length density between protonated and deuterated compounds. Here we report mild deuteration conditions utilising standard laboratory glassware for the deuteration of: diphenylamine, *N*-phenylnaphthylamine, *N*-phenyl-*o*-phenylenediamine and 1-naphthylamine (via H/D exchange in D_2_O at 80 °C, catalysed by Pt/C and Pd/C). These conditions were not successful in the deuteration of triphenylamine or *N*,*N*-dimethylaniline, suggesting that these mild conditions are not suitable for the deuteration of tertiary arylamines, but are likely to be applicable for the deuteration of other primary and secondary arylamines. The deuterated arylamines can then be used for synthesis of larger organic molecules or polymers with optoelectronic applications.

## 1. Introduction

**Figure 1:**
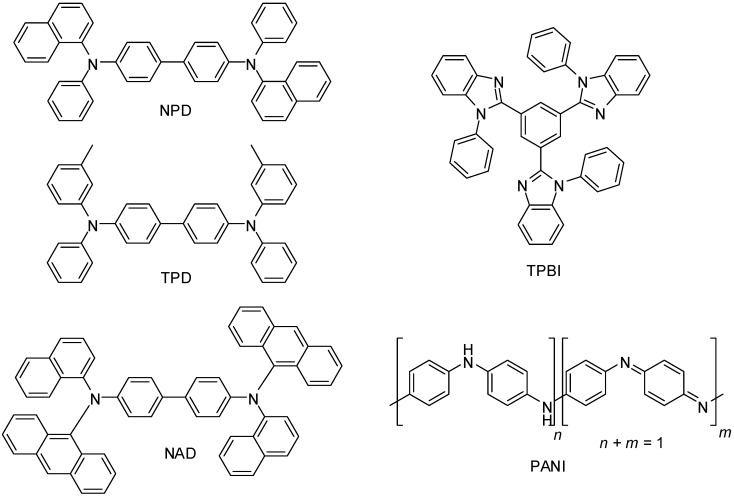
Chemical structures of compounds used for hole transport (NPD, NAD, TPD) and electron transport (TPBI) in OLED devices, and the conducting polymer polyaniline (PANI).

The discovery of the conductive properties of organic materials has led to the development of the large and expanding field of organic optoelectronics and conductive polymers, which encompasses technologies such as organic light-emitting diodes (OLED), organic photovoltaics (OPV) and dye sensitised solar cells (DSSC). In addition to their excellent electronic properties, the family of arylamines has favourable properties such as ion transfer processes, redox properties, and photoelectrochemical behaviour [1,2,3,4,5,6]. It has also been reported that arylamine compounds have strong electrochromic properties when they are oxidized [1,2,3,7]. Electron rich arylamine-based compounds (such as *N*,*N'*-di(1-naphthyl)-*N*,*N'*-diphenyl-(1,1'-biphenyl)-4,4'-diamine (NPD), *N*,*N'*-bis(3-methylphenyl)-*N*,*N'*-diphenyl-benzidine (TPD) and *N*,*N*'-di(1-naphthyl)-*N*,*N*'-diphenyl-4,4'-biphenyldiamine (NAD), Figure 1) are widely used as components in hole transport materials [8,9]. Electron deficient molecules found in OLED, such as 1,3,5-tris(*N*-phenylbenzimidizol-2-yl)benzene [10,11] (TPBI, Figure 1), are also prepared from arylamine derivatives. 

Among conducting polymers, polyaniline (PANI, Figure 1), a polymer of the simplest form of arylamine, stands out due to its favourable properties (such as air and moisture stability) and multiple applications. Polyaniline exists in three different oxidation states that have different properties (e.g. conductivity, colour), with these properties able to be exploited in sensors or electrochromic devices. Other applications for polyaniline include use in antistatic coatings [12] and hole injection layers [13]. Polymeric arylamines are also attractive due to ease of thin film fabrication via solution processing [14].

There is much interest in the development of new organic molecules with specific/tuneable and desirable electrochemical properties. Effort has been focused on improving the low electricity-to-light conversion efficiency, as well as the poor high-voltage stability that plagues OLED devices. One mechanism investigated has been the replacement of hydrogen with deuterium in the organic molecules in these devices. While it is often assumed that this substitution does not significantly change the chemical and optoelectronic properties of the parent compound, this does not take into account the relatively large difference in mass between hydrogen and deuterium, which can result in different physical properties (such as polarity and molecular volume). In a heavier isotope, its zero-point energy is lowered due to its lower potential and vibrational energy levels. These physical differences may result in deuterated molecules behaving differently in non-covalent interactions (such as π-π stacking or hydrogen bonding) compared to their protonated analogues and so it is of relevance to also study the properties of optoelectronic devices containing deuterated molecules.

It has been recently reported that selective deuteration of conducting polymers alters the optoelectronic properties of the molecules [15,16], with D-polymers showing a substantially larger magnetoresistance compared to the protonated form [16]. The deuteration of the emitting molecule tris-(8-hydroxyquinoline)aluminium (Alq_3_) resulted in an OLED device with enhanced light-emitting efficiency and high-voltage stability, compared to the respective protonated device [17]. The deuteration of tris-(2-phenylpyridine)iridium resulted in increased high-voltage stability, as well as extended device lifetime [18]. As such, there is particular interest in deuterated molecules for use in optoelectronic devices. 

This isotopic substitution is also performed in order to exploit the difference in neutron scattering length between hydrogen (−0.3742 fm) and deuterium (0.6671 fm) for neutron studies. The morphological stability of the organic layers in OLED devices can affect both the efficiency and the device total life-time [19,20,21,22,23], with heat generation during the operation of the device resulting in instability at the interfaces. Investigations of buried interfaces within functioning OLED devices presents a number of difficulties, and so neutron reflectometry has become a key method for investigations at the interfaces of thin films [8,17,24,25,26,27,28,29,30]. Selected combinations of protonated and deuterated components enhance the scattering contrast, providing the ability to probe the structures of individual components within a functional device [31,32,33].

The preparation of optoelectronic devices requires large quantities of the desired organic molecules, and so efficient synthesis of these materials is necessary, especially if deuterated molecules are required. An advantage of polymeric materials is that the polymerisation can be conducted and processed on a large scale at relatively low cost, with many of the applications (sensors, functional coatings, catalysts, *etc.*) of conducting polymers requiring large quantities of material. Therefore, developing bulk syntheses of the deuteration form of the monomers for conducting polymers would be especially important for practical reasons.

**Figure 2:**
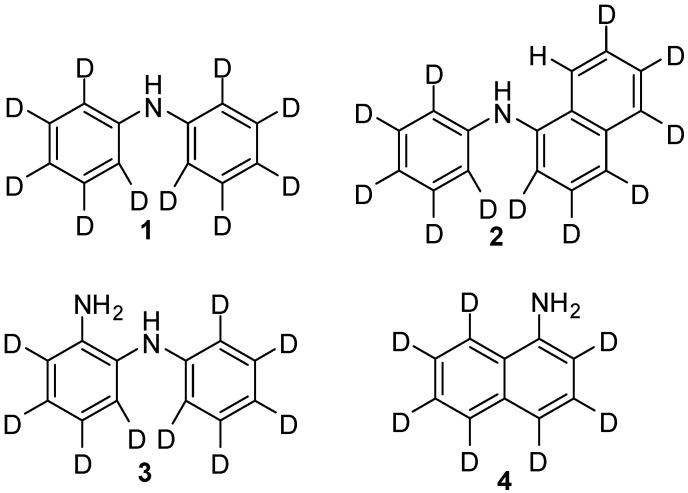
The structures of the four deuterated arylamines **1**–**4** synthesised in this study.

There are a variety of methods for performing hydrogen/deuterium-exchange reactions at carbon centres; these methods include pH-dependent exchange and exchange catalysed by either a homogeneous or heterogeneous catalyst. The source of deuterium for exchange is often D_2_O, but these reactions can also be performed using D_2_ [34] or deuterated protic solvents to provide labile deuterium atoms. Hydrothermal reaction conditions are also often necessary to achieve high levels of deuterium incorporation, however, these reactions require specialised vessels, which come at significant cost, especially for vessels capable of performing multigram synthesis [35,36,37]. Here we report a general procedure and four example compounds (**1**–**4**, Figure 2) utilising mild reaction conditions and standard laboratory glassware for the multi-gram scale deuteration of primary and secondary arylamines that are used in optoelectronic applications.

## 2. Results and Discussion

**Scheme 1:**
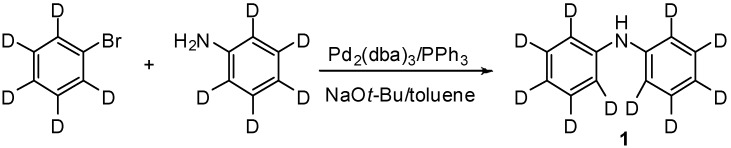
The synthesis of **1** via the Buchwald-Hartwig cross coupling of *d*_5_-bromobenzene and *d*_5_-aniline.

The synthesis of deuterated polyaromatic molecules for optoelectronic devices can be performed in a variety of ways, starting from either deuterated precursors that are assembled via standard organic chemistry techniques, or by performing hydrogen-deuterium exchange on the protonated molecule. The synthesis of three of the deuterated molecules we describe in this study (**1**, **2** and **3**, Figure 2) has been previously reported, using Buchwald-Hartwig cross coupling of the respective deuterated amines and deuterated aryl halides. For example, synthesis of **1** can be achieved by the reaction of *d*_5_-bromobenzene and *d*_5_-aniline (Scheme 1) [38], with the synthesis of **2** [38,39,40] and **3** [41] performed in a similar manner. In all cases, the deuterated starting materials are very expensive and so this method is not feasible for the large scale synthesis required for optoelectronic applications or conductive polymers. **3** has previously been synthesised via the ruthenium catalysed reaction of *d*_10_-azobenzene with isopropanol, however the desired product was only produced in 35% yield, with some undesired H-exchange also observed at positions *ortho-* to the NH_2_ [42]. Alternatively, deuteration of arylamines has been conducted using H/D exchange in D_2_O at high (>250 °C) temperature.

The H/D exchange of aniline has been performed in D_2_O (without catalyst) at 260 °C for 24 h, however, this method only reported significant deuterium exchange at the *ortho*- (47%) and *para*- (64%) positions, with only 3% exchange observed at the *meta*-position [43]. The use of near critical D_2_O (325 °C) and a polymer-supported sulfonic acid catalyst synthesised *d*_7_-aniline with 81% overall deuterium incorporation [44]. The use of a lower temperature (250 °C) and shorter reaction time (2 h) in the absence of catalyst also supported the previous finding that deuteration only occurs at the *o*- and *p*-positions, although in this case higher exchange was observed at these positions (>90% exchange) [44]. To our knowledge, H/D exchange has not been previously attempted for *N*-phenylnaphthylamine and *N*-phenyl-*o*-phenylenediamine, with H/D exchange reactions for diphenylamine and 1-naphthylamine only reported at high temperature. 

**1** has been synthesised via acid-catalysed H/D exchange of diphenylamine in D_2_O (250 °C) [45], however, this requires the specialised high-pressure equipment capable of withstanding these harsh conditions. The metal-catalysed H/D exchange of 1-naphthylamine in D_2_O utilising microwave and 150 °C has been previously used to synthesise **4** [46], however deuteration via this technique is limited by its small capacity (<100 mg). Alternatively, the nitration and subsequent reduction of *d*_8_-naphthylene has successfully yielded **4** on ~1 g scale [47], however *d*_8_-naphthylene is quite expensive, making large scale synthesis of **4** undesirable via this method. Protonated 1-naphthylamine is significantly cheaper, and so the development of mild reaction conditions for the deuteration of 1-naphthylamine would reduce costs and eliminate the need for additional synthetic steps.

It has been shown more recently that the use of a Pt/C catalyst can facilitate the H/D exchange in D_2_O of the aromatic hydrogens of aniline (and a variety of other *single*-ring aniline-type compounds, such as 1,2-diaminobenzene and 2-propylaniline) at room temperature, with increased deuterium incorporation achieved at 80 °C (up to 98% overall deuterium incorporation) in moderate (50%–60%) chemical yield [48,49]. The comparatively mild conditions facilitated by these catalysts are clearly more favourable than high temperatures (>250 °C) required for performing H/D exchange reactions in the absence of the catalysts, but these conditions have not been applied to *multi*-ring systems or *secondary* arylamines.

In this study successful deuteration of the primary and secondary arylamines **1**–**4** has been achieved on multi-gram scale using mild reaction conditions similar to those that have been used previously for aniline. The mild-deuteration of the compounds reported herein has applications in the preparation of optoelectronic devices as the molecules can be produced on larger scale, comparatively cheaply and using standard laboratory equipment. The advantages are two-fold; functioning devices containing deuterated components can be studied using neutron scattering, and the optoelectronic devices produced may also have enhanced efficiency and stability.

**Scheme 2:**
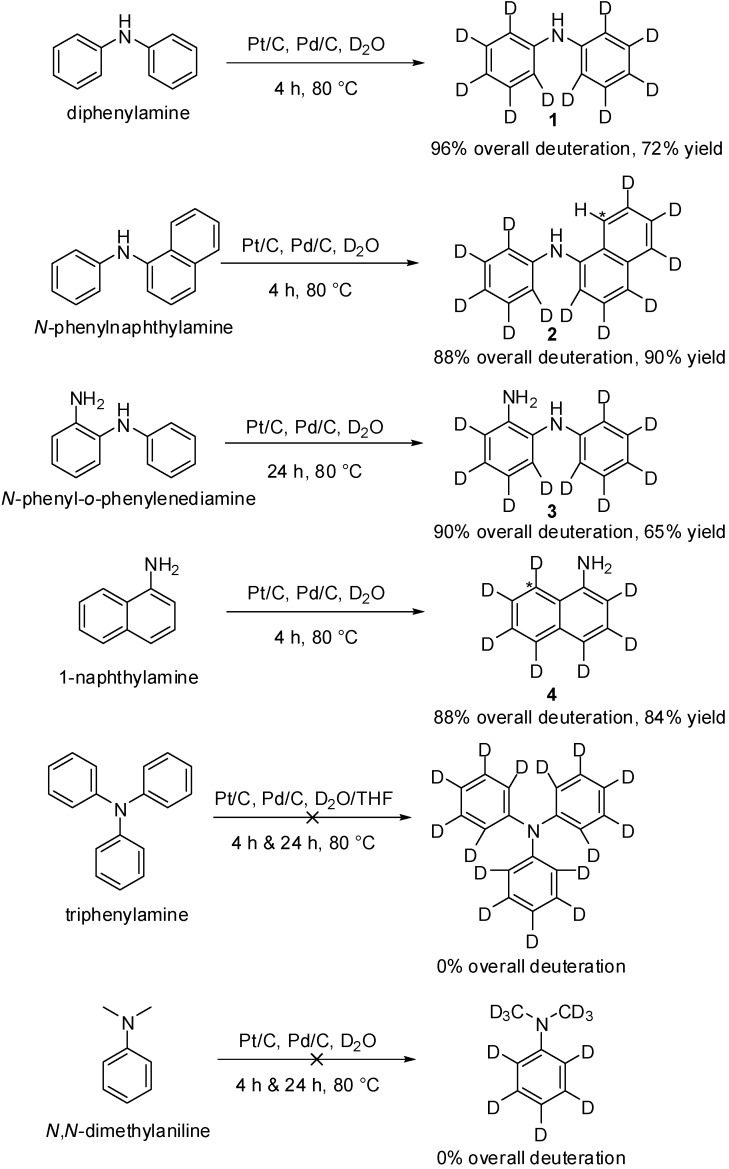
The synthesis of deuterated arylamines **1**–**4** via H/D exchange in D_2_O, catalyzed by Pt/C and Pd/C.

Here we have performed the synthesis of **1**–**4** via H/D exchange in D_2_O, facilitated by a combination of Pd/C (~1 mol %) and Pt/C (~1.5 mol %), with the reaction mixture purged with N_2(g)_ and then H_2(g)_ prior to heating at 80 °C for 4–24 h (Scheme 2). For compound **4** it was found that at longer reaction times, some saturation of the aromatic system was observed; the majority of the saturation occurred on the ring bearing the aniline moiety. Heating at 80 °C for 4 h resulted in overall deuteration of 90%, with the compound purified by recrystallisation in 84% yield, with no significant saturation observed. A 4 h reaction time was then also employed in the synthesis of compounds **1** and **2**, where no significant saturation was observed.

Compound **1** was produced in 72% yield following purification by recrystallisation, with 96% overall deuteration achieved. Compound **2** was produced in 90% yield, with 88% overall deuteration achieved. One position on **2** (marked by * on **2** in Scheme 2) appeared to undergo no H/D exchange as observed in the ^1^H- and ^2^H-NMR spectra, which contributed to the lower overall deuteration level. It is likely that the lack of H/D exchange at this position is due to a combination of steric crowding from the phenyl ring and hydrogen bonding with the amine group. Incomplete deuteration (to a lesser extent) of the analogous position was observed in compound **4** (marked with * in Scheme 2), where H-bonding with the amine group is the contributing factor. Compound **3** required a longer reaction time of 24 h to achieve 90% overall deuteration with 65% yield after column chromatography; only ~60% overall deuteration was observed at 4 h. No saturation was observed after 24 h. The need for longer reaction time for the synthesis of **3** was likely attributed to the higher melting point (77–80 °C) of this solid compared to **1** (50–53 °C), **2** (60–66 °C) and **4** (47–50 °C). High melting point compounds are likely to have lower miscibility and solubility in D_2_O at 80 °C and therefore H/D exchange rate is likely to be affected. It should be noted that higher overall deuteration levels for these compounds can be achieved by returning the compound for a second cycle with fresh D_2_O and catalyst, however, for the scope of this study single H/D exchange cycle was sufficient.

The deuteration of the tertiary arylamine triphenylamine was attempted using the same method (Scheme 2). After 4 h at 80 °C, no deuteration was observed (as measured by mass spectroscopy), and so THF was added to enhance the solubility of triphenylamine (m.p. 127 °C). The reaction continued at 80 °C for a further 24 h, however, no deuteration was observed. This is not surprising, as the literature reports H/D exchange of triphenylamine in D_2_O with platinum(IV) oxide catalyst at 250 °C for 12 h [50]. Deuteration of the tertiary arylamine *N*,*N*-dimethylaniline, which has a much lower melting point, 2 °C, was also attempted using this method (Scheme 2), with no deuteration observed at either 4 or 24 h. This led us to generally conclude that the mild deuteration conditions presented here are only applicable to primary and secondary arylamines.

It is apparent that the availability of the nitrogen lone pair influences the deuteration reaction. The basicity of arylamine is reduced dramatically when moving from primary (p*K*_a_ = ~4.6) and secondary (p*K*_a_ = ~0.8) to tertiary (p*K*_a_ = ~−5). It is expected that the metal catalyst can be located in the vicinity of the nitrogen atom of primary and secondary arylamines more so than that in the tertiary arylamine since Pt and Pd have a quite high affinity for the nitrogen lone pair. This could be the reason why no deuteration was observed in triphenylamine. Another possible explanation for this behaviour would be the ability of the nitrogen lone pair in primary and secondary arylamines to form hydrogen bonding with water (D_2_O), which enhances their miscibility and solubility at 80 °C. However the unsuccessful deuteration of the liquid compound *N*,*N*-dimethylaniline suggests that miscibility is not the main driving factor for the deuteration that is observed in the primary and secondary arylamines examples used in this study. 

**Scheme 3:**
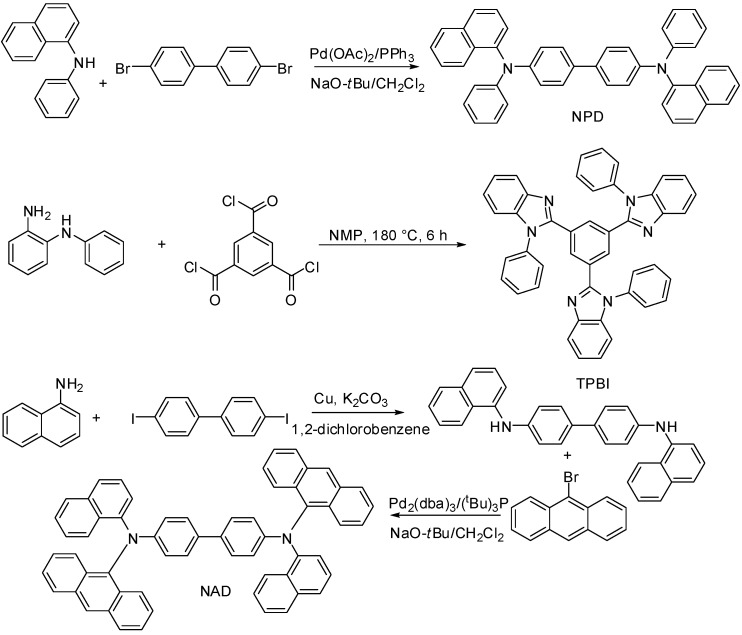
Utility of the deuterated compounds described in this study for the synthesis of organic molecules (NPD [51], TPBi [10] and NAD [52]) with photoelectronic applications.

The deuterated arylamines **1**–**4** can then be used for the synthesis of a variety of compounds with photoelectronic applications, where direct deuteration of the final molecule is often not achievable due to low solubility. For example, **2** can be used in the synthesis of *d*-NPD, **3** can be used in the synthesis of *d*-TPBI, and **4** can be used in the synthesis of *d*-NAD (Scheme 3), using analogous methods to those reported in the literature for the synthesis of the protonated compounds [10,51,52]. Loss of deuteration *via* back-exchange is minimized due to the absence of protic solvents, indeed we did not observe any back exchange in the synthesis of *d*-tris(4-carbazoyl-9-ylphenyl)amine (*d*-TCTA) from deuterated carbazole [33]; this method is similar to those used for the synthesis of the compounds described in Scheme 3.

## 3. Experimental Section 

### 3.1. Materials

Diphenylamine, *N*-phenyl-*o*-phenylenediamine, 1-naphthylamine, *N*-phenylnaphthylamine, triphenylamine, *N*,*N*-dimethylaniline, platinum on carbon (10%), palladium on carbon (10%) and Celite (545, reagent grade) were purchased from Sigma Aldrich (Sydney, Australia). D_2_O (99.8%) was purchased from AECL, Ontario, Canada. NMR solvents were purchased from Cambridge Isotope Laboratories, Inc. (Tewksbury, MA, USA) or Sigma Aldrich. 

### 3.2. Instrumentation

^1^H-NMR (400 MHz), ^13^C-NMR (100.6 MHz) and ^2^H-NMR (61.4 MHz) spectra were recorded on a Bruker 400 MHz spectrometer at 298 K. Chemical shifts, in parts per million, were referenced to the residual signal of the corresponding NMR solvent. Deuterium NMR was performed using the probe’s lock channel for direct observation. Electrospray ionization mass spectra (ESI-MS) were recorded on a 4000 QTrap AB Sciex spectrometer. The overall percentage deuteration of the molecules was calculated by MS using the isotope distribution analysis of the different isotopologues. This was calculated taking into consideration the ^13^C natural abundance (as determined from the mass spectrum of the protonated material), whose contribution was subtracted from the peak area of each [M+1] isotopologue to allow for accurate estimation of the percentage deuteration. The mean molecular weight was calculated based on the overall percentage of deuteration, and this was used for the calculation of mass yields. Automated medium pressure chromatography was conducted using a Reveleris^®^ flash chromatography system (Grace Davison, Rowville, VIC, Australia), with specific chromatographic conditions given as details for the relevant compound.

### 3.3. General Method

The relevant protonated arylamine (1–12 g) was suspended in D_2_O (~25 mL per gram of arylamine) in a two-neck round bottom flask, to which Pt/C (10% w/w, ~1 mol %) and Pd/C (10% w/w, ~1.5 mol %) were added. The flask was sealed, and the stirring reaction mixture was purged with one balloon of N_2(g)_, followed by one balloon of H_2(g)_, ensuring that the flask was not pressurised. The reaction mixture was heated at 80 °C for 4 h in the case of **1**, **2** and **4**, and 24 h in the case of **3**. The reaction mixture was allowed to cool, CH_2_Cl_2_ (~25 mL) was added to the reaction flask, and the mixture filtered over Celite to remove the catalyst, which was then washed with CH_2_Cl_2_ (50–100 mL). The filtrate was extracted with 3 × 50 mL CH_2_Cl_2_, the combined organics dried over Na_2_SO_4_, filtered and evaporated to dryness to yield the deuterated product, characterized as follows:

*d_10_-Diphenylamine* (**1**). Compound **1** was synthesized according to the general procedure, utilizing 12 g of diphenylamine, 2 g of Pt/C and 2 g of Pd/C. The product was recrystallised from petroleum ether to give cream coloured crystals (9.1g, 72%). ^1^H-NMR (400 MHz, *d*_6_-acetone): δ residual protons 7.36 (br s, 1H), 7.24 (s, 0.24H), 7.12 (s, 0.07H), 6.85 (s, 0.04H). ^2^H-NMR (61.4 MHz, *d*_6_-acetone): δ 7.28 (br), 7.17 (br), 6.89 (br). ^13^C-NMR (101 MHz, *d*_6_-acetone): δ 144.5 (m), 129.5 (m), 120.4 (m), 117.6 (m). ESI-MS *m/z*: 180.1 [M+H]^+^. Overall 95.8% D levels with isotopic distribution *d*_8_ 6.3%, *d*_9_ 29.9%, *d*_10_ 63.9%.

*d_12_-N-Phenylnaphthylamine* (**2**). Compound **2** was synthesized according to the general procedure, utilising 6 g of *N*-phenylnaphthylamine, 1 g of Pt/C and 1 g of Pd/C. No additional purification was necessary, giving the product as a beige coloured solid (5.7 g, 90%). ^1^H-NMR (400 MHz, *d*_6_-acetone): δ residual protons 8.18 (s, 1H), 7.89 (s, 0.05H), 7.56 (s, 0.06H), 7.48 (br s, 1H), 7.41 (s, 0.05H), 7.39 (s, 0.05H), 7.23 (s, 0.08H), 7.08 (s, 0.08H), 6.85 (s, 0.04H). ^2^H-NMR (61.4 MHz, *d*_6_-acetone): δ 7.94 (br), 7.67–7.36 (m), 7.28 (br), 7.13 (br), 6.90 (br). ^13^C NMR (101 MHz, *d*_6_-acetone): δ 146.1 (s), 140.4 (s), 135.7 (s), 129.4 (m), 128.6 (m), 128.5 (s), 126.4 (m), 126.3 (m), 125.6 (m), 123.2 (s, C-H*), 122.4 (m), 120.1 (m), 117.7 (m), 115.3 (m). ESI-MS *m/z*: 231.1 [M+H]^+^ overall 87.8% D levels with isotopic distribution *d*_7_ 1.3%, *d*_8_ 2.6%, *d*_9_ 7.8%, *d*_10_ 27.2%, *d*_11_ 51.5%, *d*_12_ 9.4%.

*d_9_-N-Phenyl-o-phenylenediamine* (**3**). Compound **3** was synthesized according to the general procedure, utilising 1 g of *N*-phenyl-*o*-phenylenediamine, 0.2 g of Pt/C and 0.2 g of Pd/C. The reaction was heated for 24 h, with the standard work up employed. The product was purified using automated medium pressure liquid chromatography (40 g silica column) with petroleum ether/ethyl acetate (80:20) as mobile phase to yield a red/brown solid (0.65 g, 62%). ^1^H-NMR (400 MHz, *d*_6_-acetone): δ residual protons 7.13 (s, 0.08 H), 7.06 (s, 0.04H), 6.91 (s, 0.04H), 6.82 (s, 0.04H), 6.75 (s, 0.07H), 6.70 (s, 0.04H), 6.61 (s, 0.04H), 6.43 (br s, 1H), 4.42 (br s, 2H). ^2^H-NMR (61.4 MHz, *d*_6_-acetone): δ 7.17 (br), 7.10 (br), 6.95 (br), 6.86 (br), 6.79 (br), 6.66 (br). ^13^C-NMR (101 MHz, *d*_6_-acetone): δ 146.5 (s), 143.2 (s), 128.5 (m), 128.2 (s), 124.7 (m), 124.3 (m), 117.7 (m), 117.0 (m), 115.2 (m), 114.5 (m). ESI-MS *m/z*: 194.1 [M+H]^+^. Overall 90.1% D levels with isotopic distribution *d*_0_ 2.0%, *d*_5_ 1.8%, *d*_6_ 2.5%, *d*_7_ 7.9%, *d*_8_ 35.9%, *d*_9_ 50.1%.

*d_7_-1-Naphthylamine* (**4**). Compound **4** was synthesised according to the general procedure, utilising 4 g of 1-naphthylamine, 0.5 g of Pt/C and 0.5g of Pd/C. After heating for 4 h and cooling to room temperature, the reaction mixture was basified by addition of 28% NH_3(aq)_ (20 mL) before the standard work up. The product was purified by recrystallization from petroleum ether to give needle type brown coloured crystals (3.5 g, 83.5%). ^1^H-NMR (400 MHz, CD_2_Cl_2_): δ residual protons 7.86 (s, 0.27H), 7.50 (m, 0.08H), 7.34 (m, 0.09H), 6.81 (s, 0.04H), 4.21 (br s, 2H). ^2^H-NMR (61.4 MHz, CD_2_Cl_2_): δ 7.89 (br), 7.54 (br), 7.39 (br), 6.85 (br). ^13^C-NMR (101 MHz, CD_2_Cl_2_): δ 142.0 (s), 134.3 (s), 128.1 (m), 125.6 (m), 125.4 (m), 124.4 (m), 123.6 (s), 120.8 (s, C-H*), 120.4 (m), 118.5 (m), 109.3 (m). ESI-MS *m/z*: 151.2 [M+H]^+^ overall 88.0% D levels with isotopic distribution *d*_3_ 0.7%, *d*_4_ 2.4%, *d*_5_ 10.7%, *d*_6_ 29.9%, *d*_7_ 44.2%. 

## 4. Conclusions

This work describes the H/D exchange of primary and secondary arylamines, performed in D_2_O at 80 °C utilising Pt/C and Pd/C catalysts. These mild reaction conditions do not require any specialised laboratory equipment and can produce deuterated arylamines in multi-gram quantities, with high (>90%) levels of overall deuteration. The deuteration of organic molecules within optoelectronic devices provides opportunities to study the functioning devices using neutron reflectometry, which is able to exploit the scattering length differences between hydrogen and deuterium. These deuterated arylamines can also be incorporated into optoelectronic devices, which may have better lifetime and efficiency compared to the protonated version. 

## Supplementary Materials

^1^H-NMR, ^2^H-NMR and ^13^C-NMR spectra, as well as mass spectra are available for compounds **1**–**4** in the supplementary material. Supplementary materials can be accessed at: http://www.mdpi.com/1420-3049/19/11/18604/s1.

## Supplementary Files

Supplementary File 1
