# Supplementary material for: Mild Conditions for Deuteration of Primary and Secondary Arylamines for the Synthesis of Deuterated Optoelectronic Organic Molecules
Source: Molecules. 2014 Nov 13;19(11):18604–17. doi: 10.3390/molecules191118604 (PMC6271713; doi:10.3390/molecules191118604)

Supplementary Materials

Figure S1. ¹H-NMR spectrum of **1** (400 MHz, d_6-acetone).

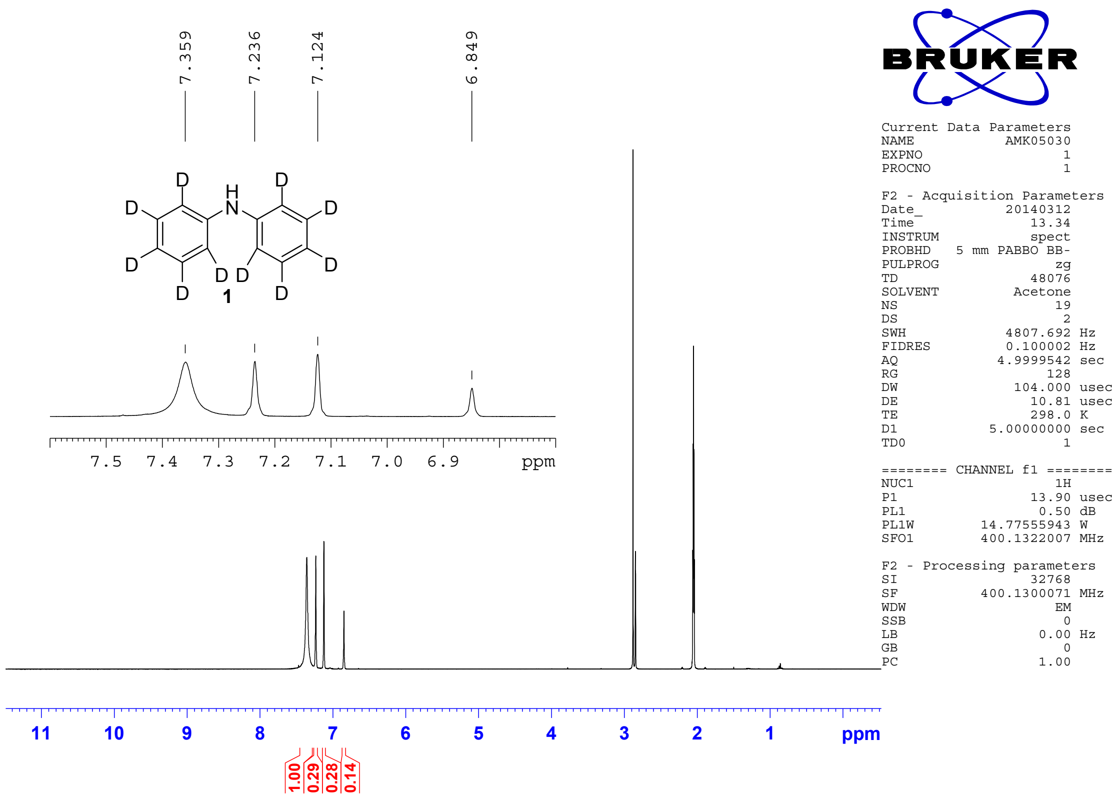

Figure S2. ²H-NMR spectrum of **1** (61.4 MHz, d_6-acetone).

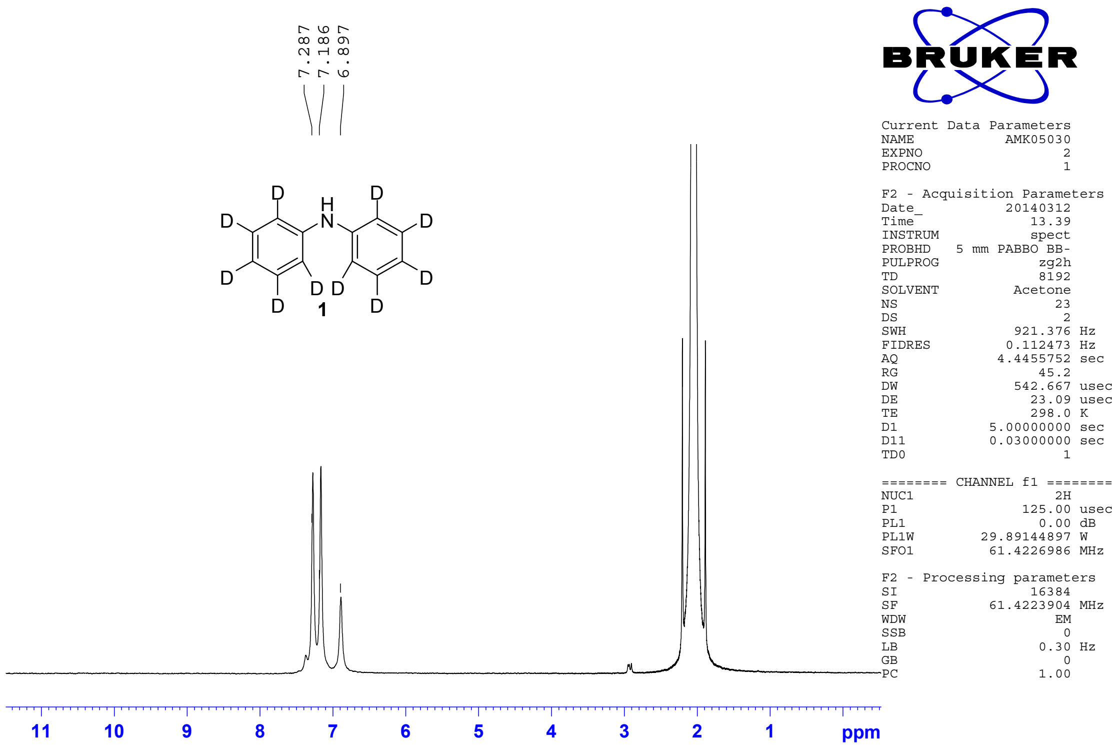

Figure S3. ^{13}C-NMR spectrum of **1** (101 MHz, d_6-acetone).

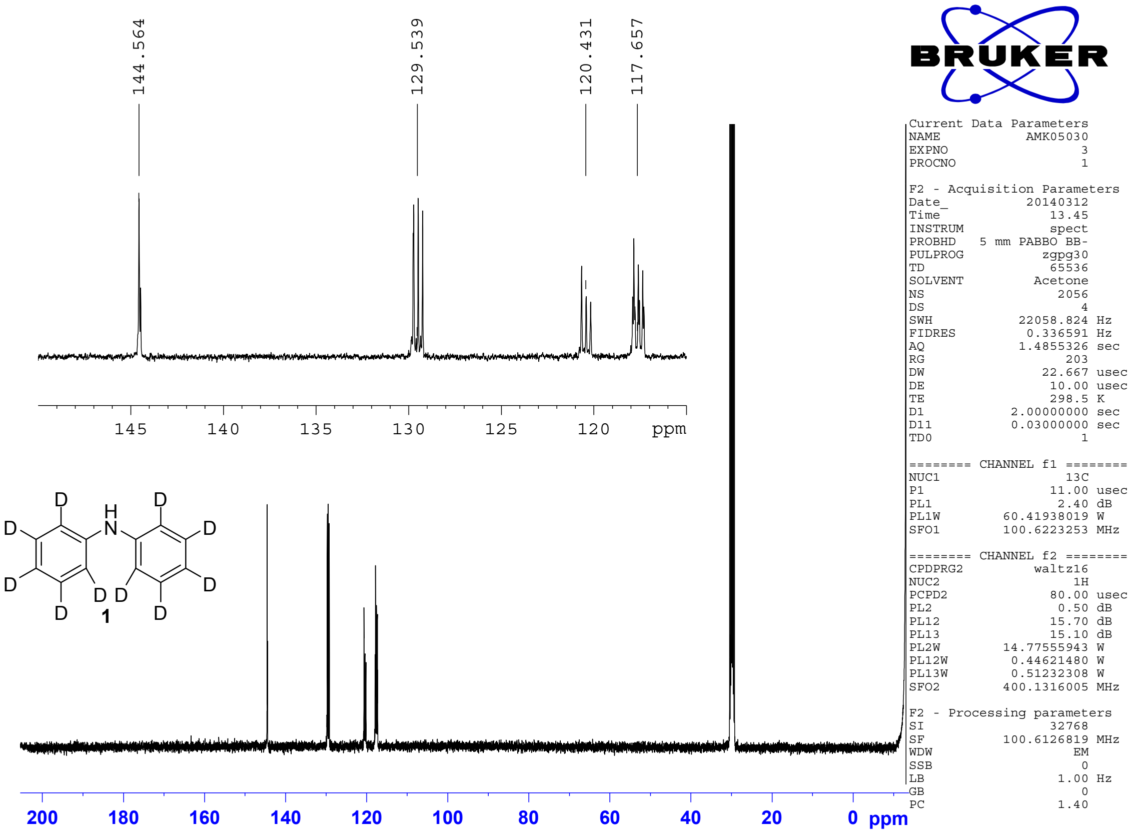

Figure S4. Enhanced resolution (ER +) mass spectrum of **1**.

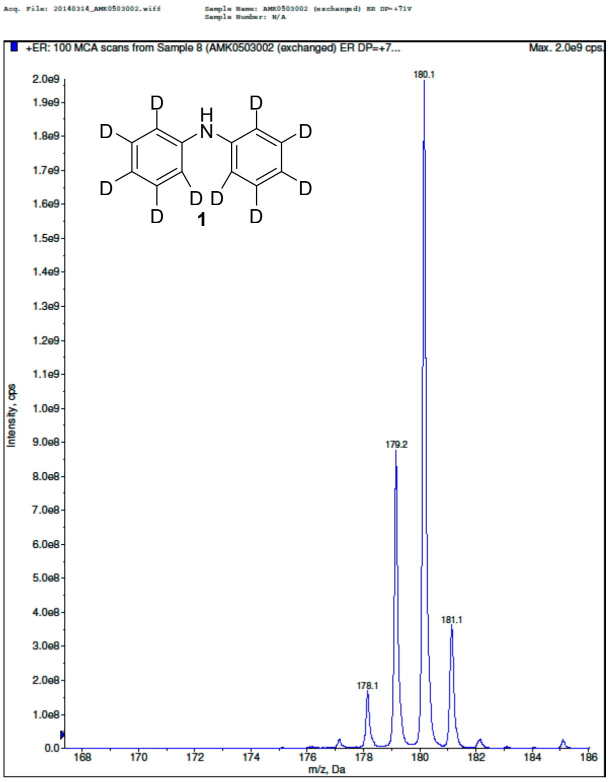

Figure S5. ^{1}H-NMR spectrum of **2** (400 MHz, d_6-acetone).

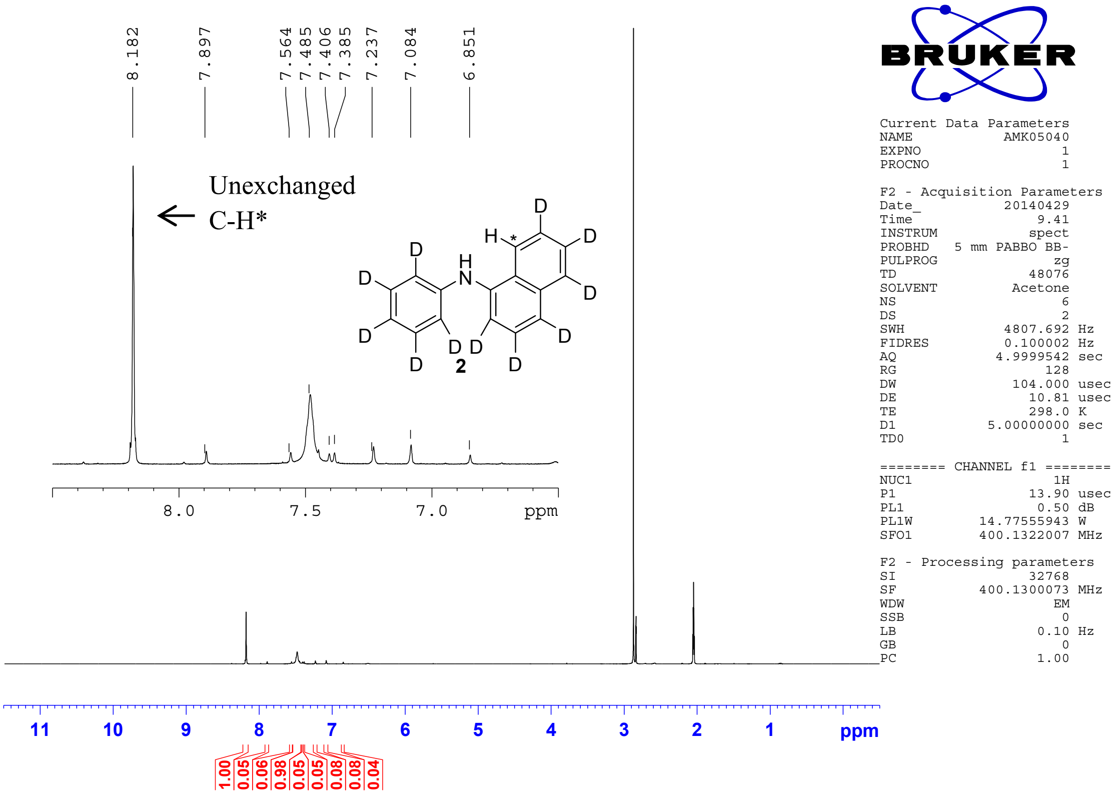

Figure S6. ^{2}H-NMR spectrum of **2** (61.4 MHz, d_6-acetone).

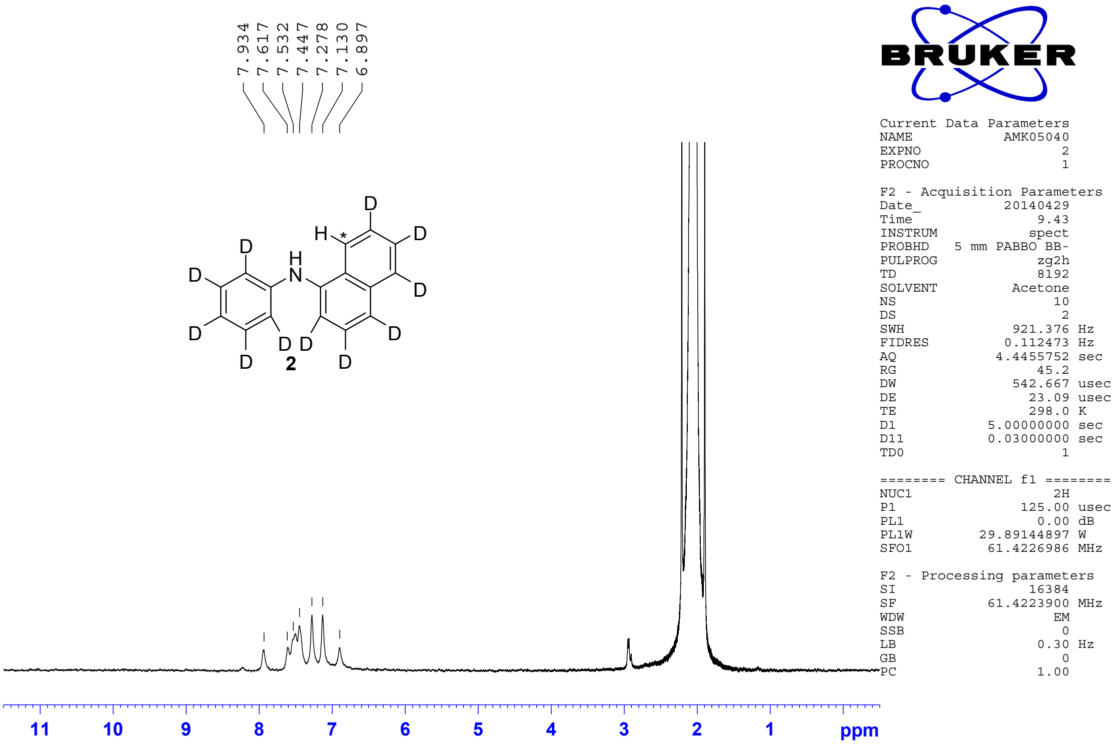

Figure S7. ^{13}C-NMR spectrum of **2** (101 MHz, d_6-acetone).

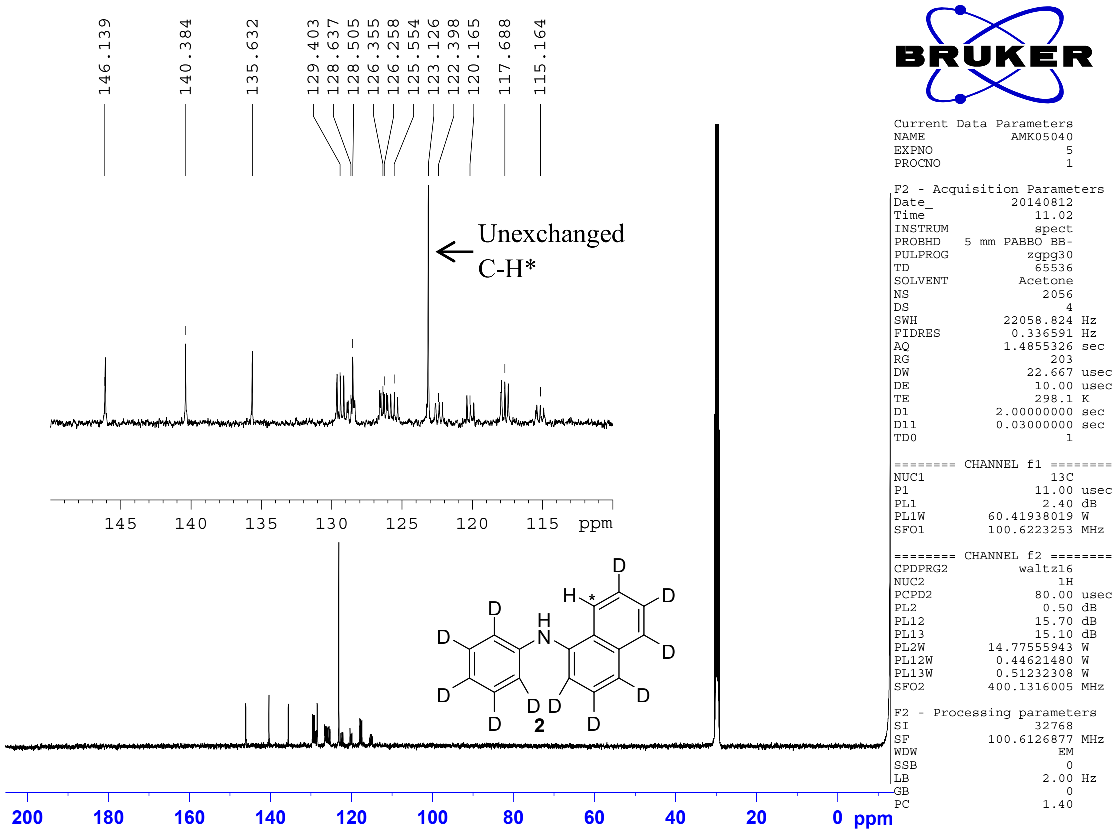

Figure S8. Enhanced resolution (ER+) mass spectrum of **2**.

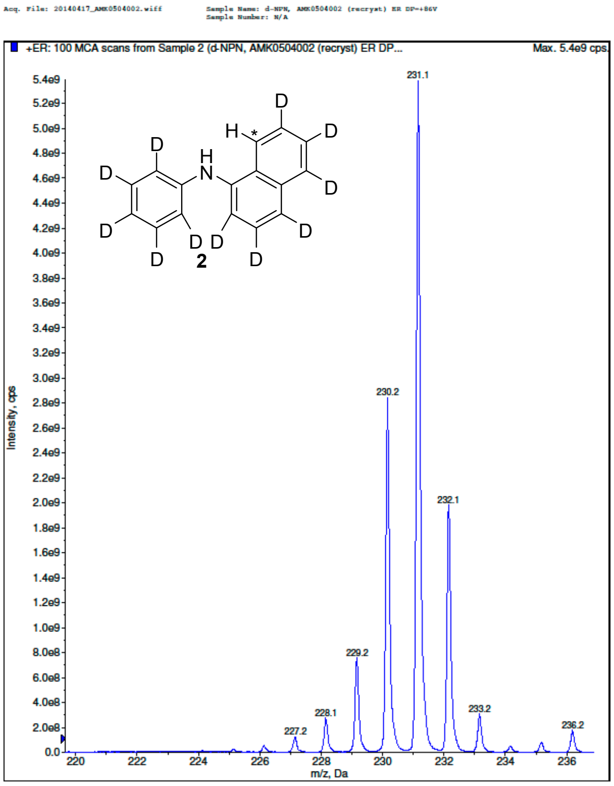

Figure S9. ^{1}H-NMR spectrum of **3** (400 MHz, d_6-acetone).

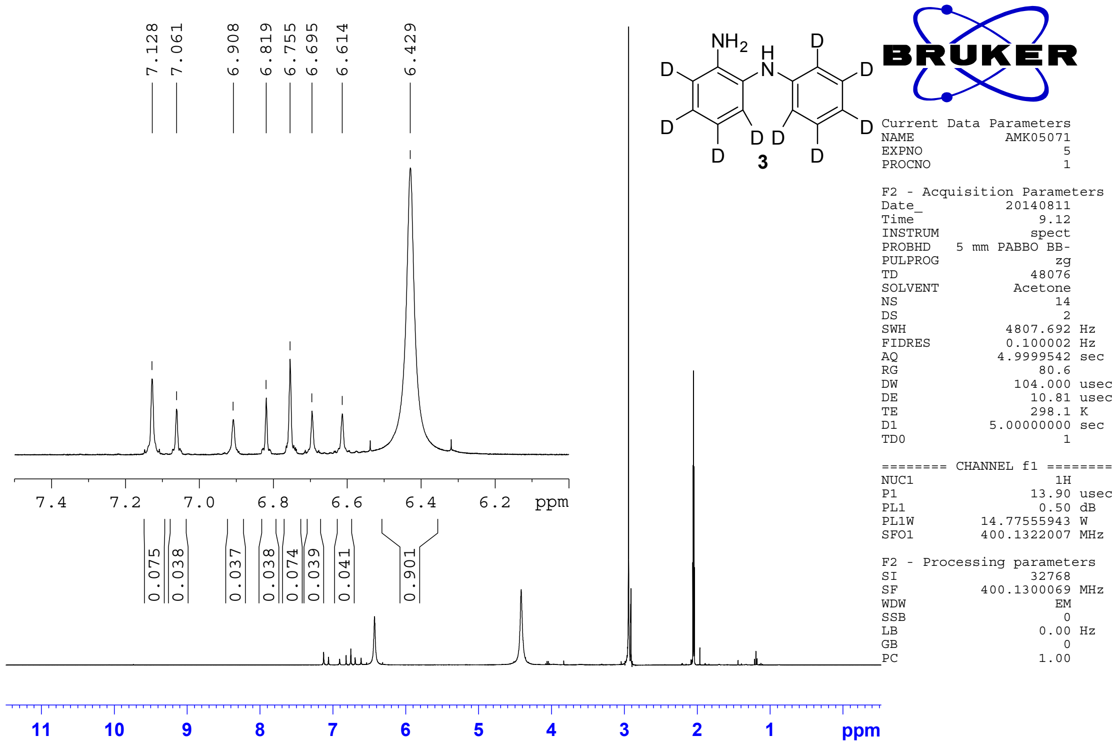

Figure S10. ^{2}H-NMR spectrum of **3** (61.4 MHz, d_6-acetone).

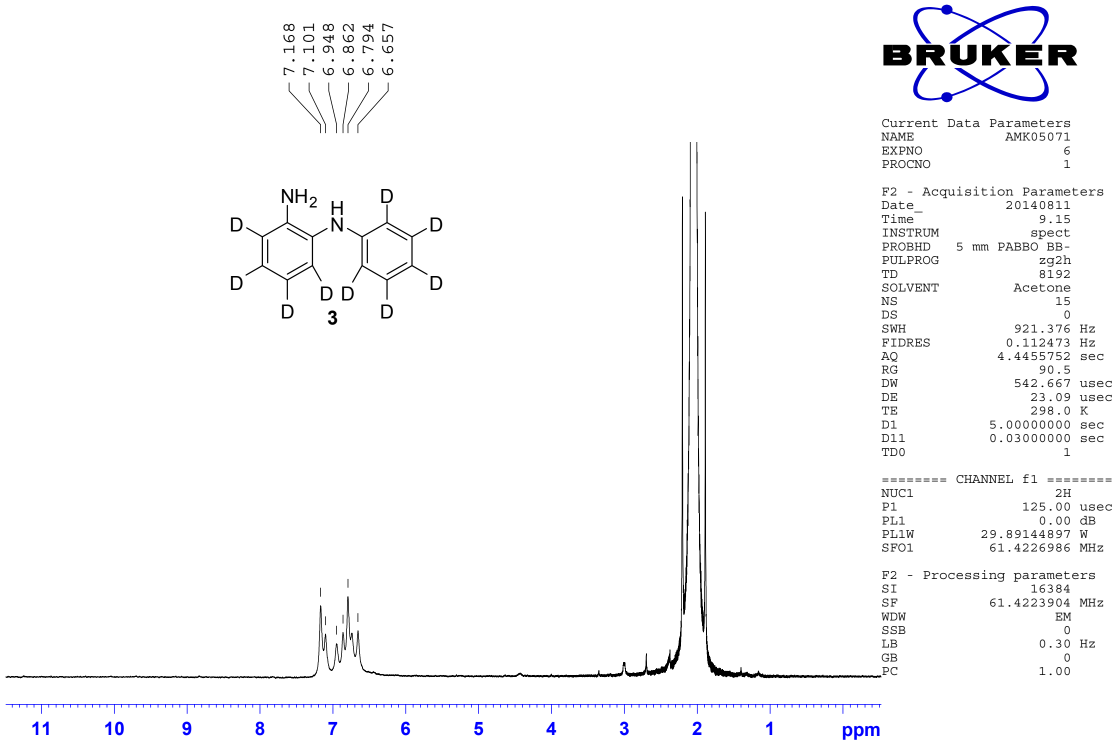

Figure S11. ^{13}C-NMR spectrum of **3** (101 MHz, *d*$_6$-acetone).

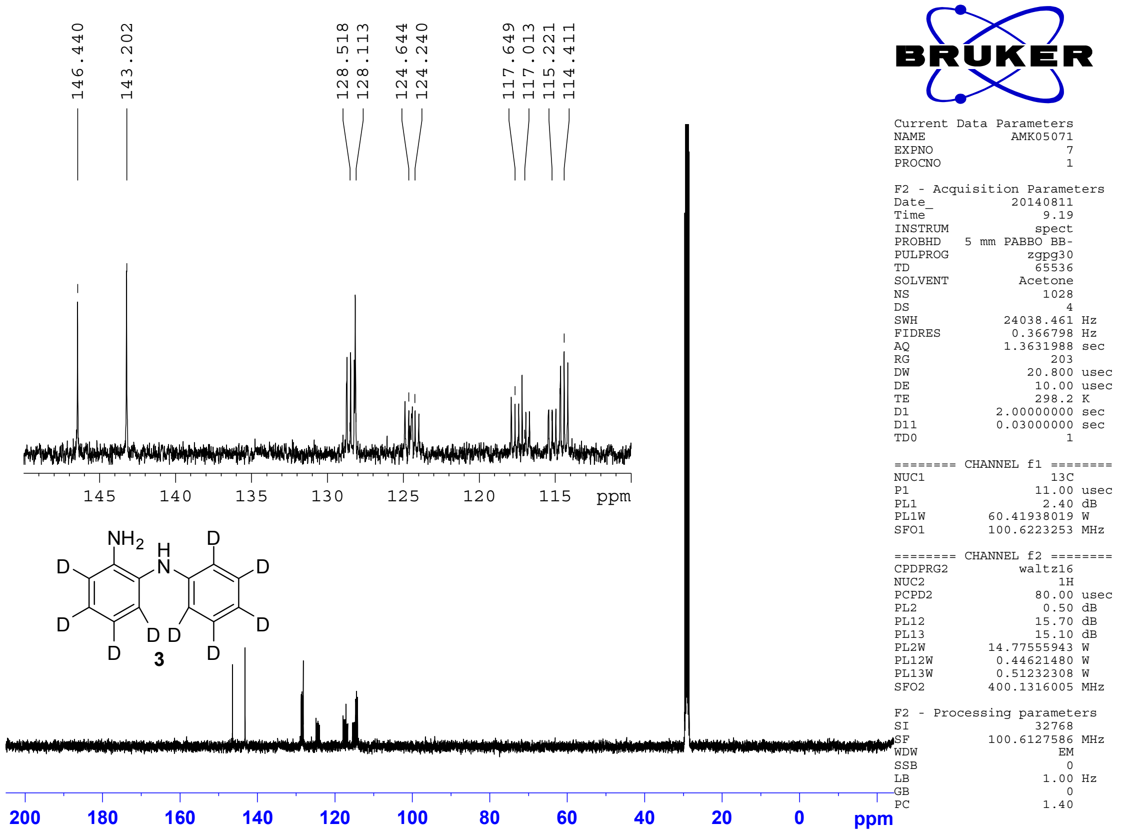

Figure S12. Enhanced resolution (ER+) mass spectrum of **3**.

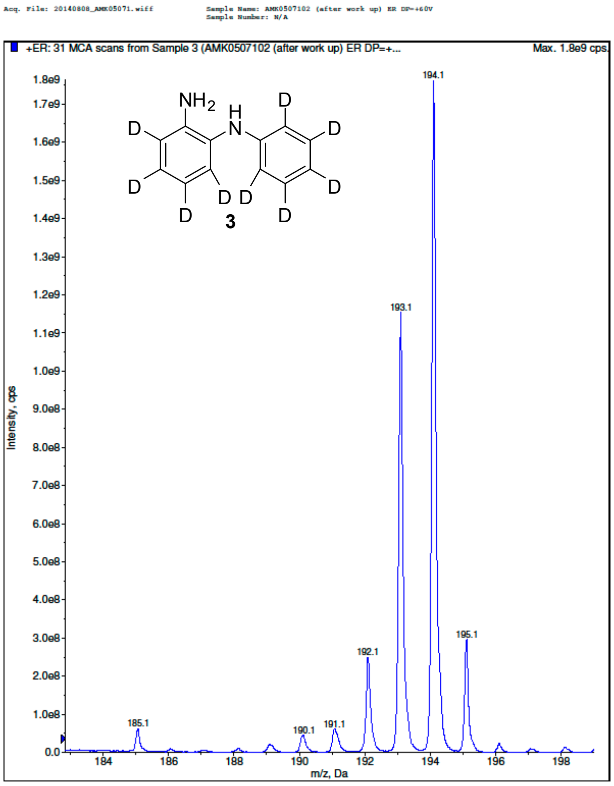

Figure S13. ^{1}H-NMR spectrum of **4** (400 MHz, CD_2Cl_2).

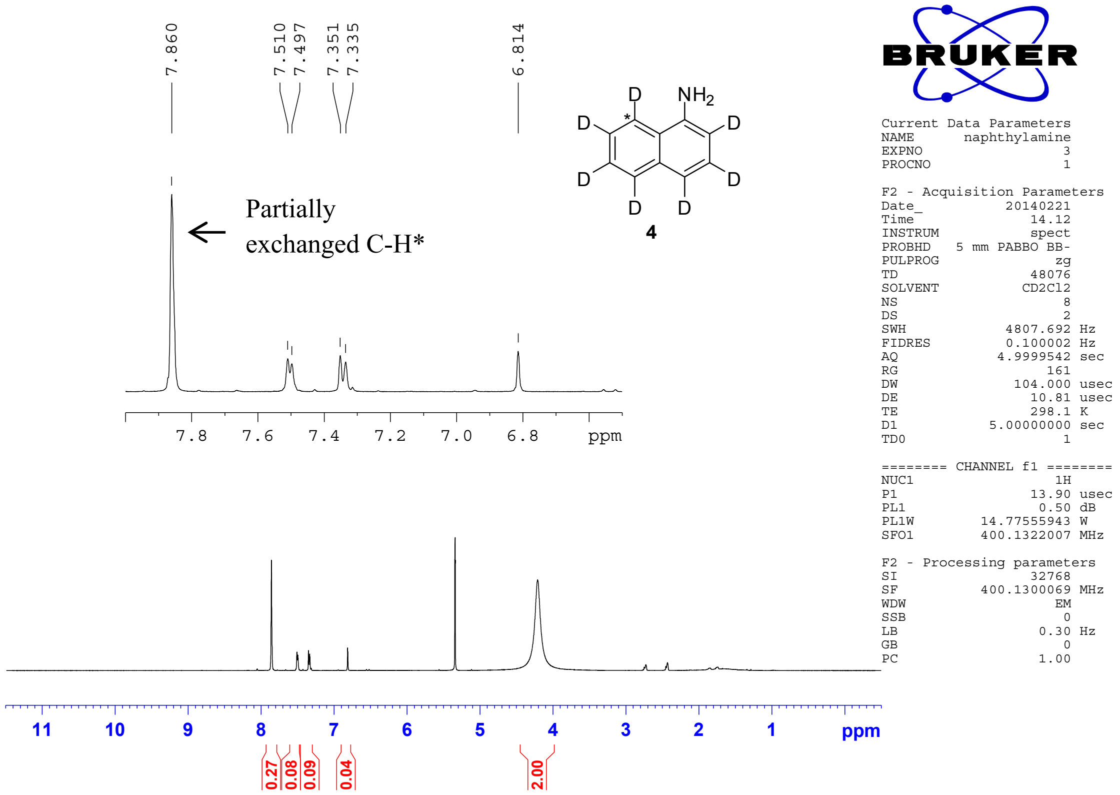

Figure S14. ^{2}H-NMR spectrum of **4** (61.4 MHz, CD_2Cl_2).

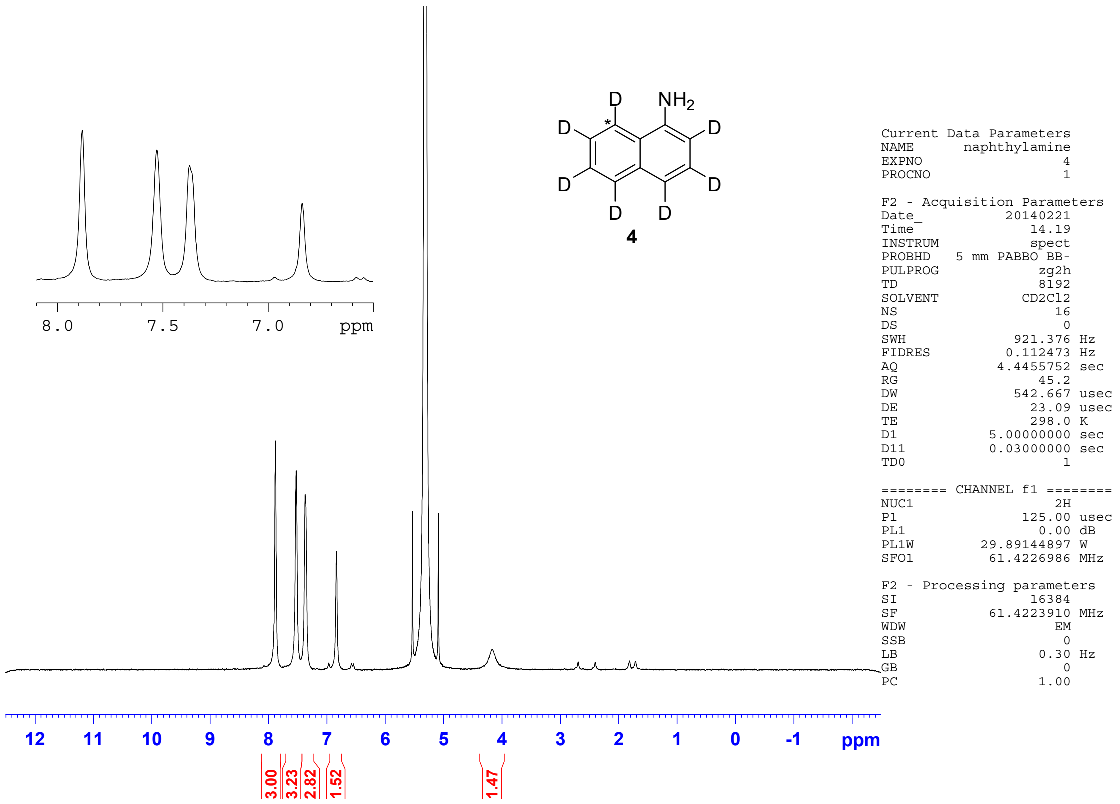

Figure S15. ^{13}C-NMR spectrum of **4** (100 MHz, CD_2Cl_2). Partial deuteration occurred at the position marked *, which shows a singlet at 120.8 ppm and an isotopically shifted triplet upfield at 120.4 ppm.

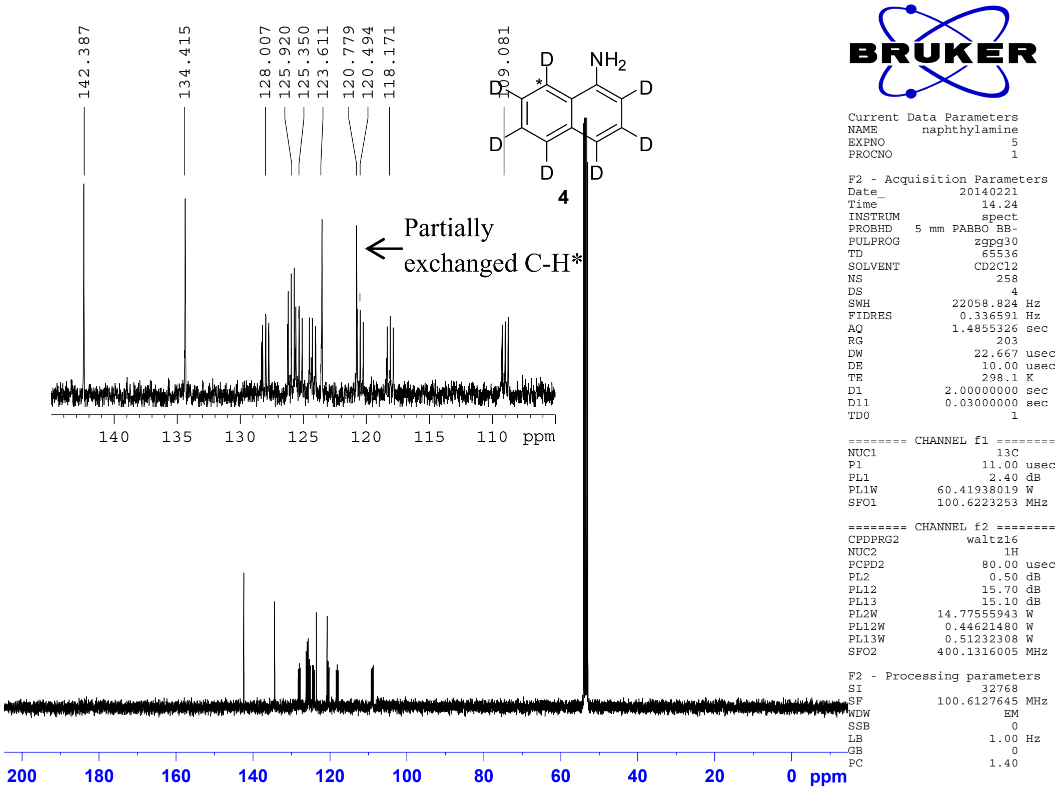

Figure S16. Enhanced mass spectrum (EMS+) of **4**.

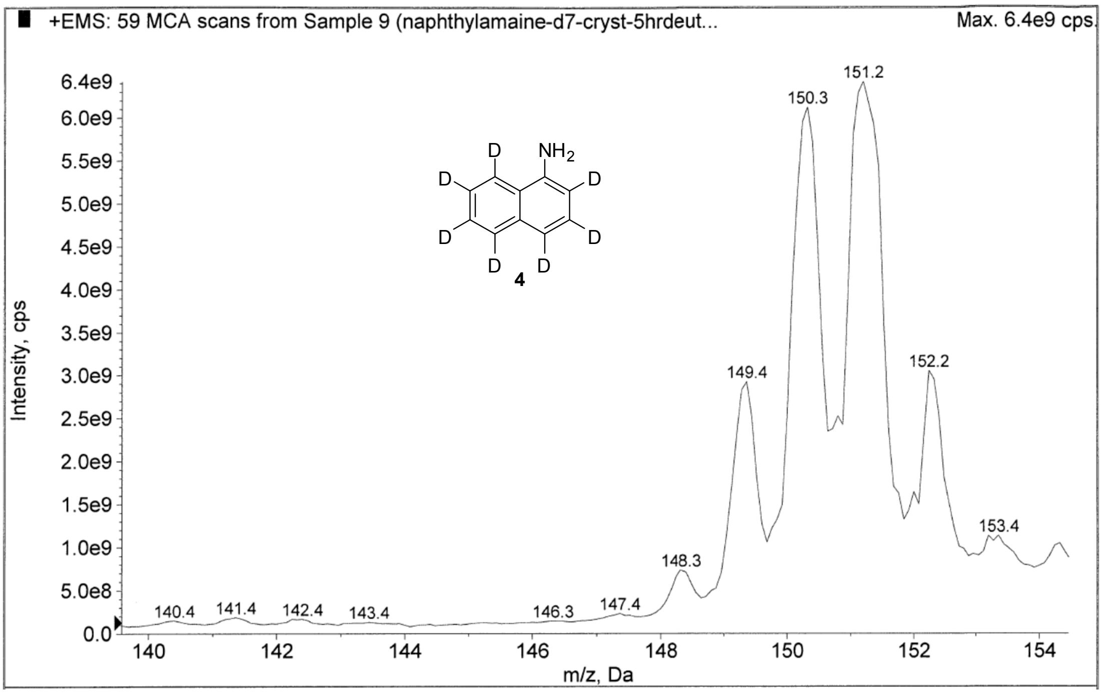

Supplement: Supplementary File 1 [file molecules-19-18604-s001.pdf]
